# An aggressive, unresected pineoblastoma in an adult woman: the role of exclusive radiotherapy – a case report and literature review

**DOI:** 10.3332/ecancer.2025.1909

**Published:** 2025-05-15

**Authors:** Salem Ouaddane Alami, Fatima Zahra Abdelli, Samia Khalfi, Fatima Zahra Farhane, Zineb Alami, Touria Bouhafa

**Affiliations:** Radiotherapy Department, Oncology Hospital, Hassan II University Hospital, Fes 30050, Morocco

**Keywords:** pineoblastoma, adult, surgery, radiotherapy, chemotherapy

## Abstract

**Introduction:**

The pineal gland is a small, endocrine structure located in the posterior cranial fossa, playing a critical role in regulating sleep-wake cycles. Pineal parenchymal tumours (PPTs) are rare, accounting for less than 1% of central nervous system malignancies. These tumours include pineocytomas (benign), intermediate-grade tumours (PPTs) and aggressive pineoblastomas (PBL), which represent the most dangerous subtype. PBL are fast-growing, high-grade tumours often classified as grade IV. This paper presents a rare case of unresected PBL in a young woman, leading to bilateral blindness and the role of exclusive radiotherapy (RT) as a treatment modality.

**Objective:**

This case report aims to explore the effectiveness of exclusive RT in treating an unresected PBL in a young patient, emphasising the tumour’s aggressiveness and the therapeutic challenges it presents.

**Case presentation:**

A 35-year-old woman with no significant medical history presented with progressively worsening headaches and visual acuity loss. Imaging revealed a pineal region tumour, confirmed as PBL through stereotactic biopsy. The patient had bilateral papilledema and developed hydrocephalus due to tumour growth, leading to pressure on surrounding structures. Due to the patient’s refusal of chemotherapy (CT), RT was chosen as the sole treatment option.

RT was delivered in two phases: a craniospinal dose of 36 Gy and a boost of 18 Gy to the tumour. The patient tolerated the treatment well, with only mild nausea and fatigue. Follow-up imaging at 3 months showed a 38% reduction in tumour size and improvement in hydrocephalus, although the patient remained bilaterally blind. At 24 months post-treatment, the tumour remained stable, suggesting a positive long-term outcome with exclusive RT.

**Discussion:**

PBL are aggressive tumours with a high potential for leptomeningeal dissemination. Surgery, when feasible, is considered the primary treatment for pediatric cases, but in adults, data are sparse. In this case, the patient’s refusal of CT made RT the only viable option. Recent studies suggest that RT alone can improve survival, particularly when combined with craniospinal irradiation and tumour boosts. However, the role of surgery and CT in adult PBL remains debated, with mixed results in the literature regarding their impact on overall survival.

While some studies indicate no significant survival benefit from extensive tumour resection, others suggest that total resection can improve prognosis. The case highlights that exclusive RT can be a life-saving treatment for patients who cannot undergo surgery or CT, despite the tumour’s aggressive nature. Further research is needed to establish standardised protocols for managing adult PBL.

**Conclusion:**

This case demonstrates the potential of exclusive RT as an effective treatment for PBL in patients who cannot undergo surgery. Despite the patient’s permanent blindness, RT led to a significant reduction in tumour size and stabilisation of the disease. More research is necessary to define the most effective treatment strategies for PBL, particularly in adults, where surgical options may be limited.

## Introduction

The pineal gland is an endocrine gland located in the posterior cranial fossa of the brain. Its importance lies in the circadian cycle of sleep and wakefulness. The pineal gland is also known as the cerebral epiphysis [1].

Pineal parenchymal tumours (PPT) account for less than 1% of malignant tumours of the central nervous system [2].

Pineal gland tumours can be of one of the following types: Pineocytomas tend to have a good prognosis, PPTs and pineal papillary tumours, which are of intermediate grade (grades II or III). And finally, pineoblastoma (PBL), a very rare, aggressive and fast-growing tumour (grade IV) [3].

Recent molecular characterisation has separated Pinealoblastoma (PB) into five molecular subgroups: PB-Group 1, PB-Group 2, PB-Group 3, retinoblastoma (RB) and MYC; each with distinct clinico-pathological and survival characteristics [12, 13]. PB Groups 1–3, appear in older children and adolescents, are associated with better outcomes, in contrast to patients in the RB and MYC groups [13].

In this paper, we report a case of unresected PBL responsible for bilateral blindness in a young woman.

## Objective

The aim of this case report is to examine the role of exclusive radiotherapy (RT) in the curative treatment of an unresected PBL in a young patient.

## Case presentation

Our patient is a 35-year-old woman, married and mother of four children, with no notable pathological medical history. She is the spouse of a patient being followed for nasopharyngeal carcinoma. She was admitted for the management of a PBL, with a history dating back approximately 2 months prior to her admission. Her symptoms included a progressive onset of headaches and decreased visual acuity. This prompted a consultation with an ophthalmologist, followed by neuroimaging, which revealed a unresctable tumour process. The case was discussed in a multidisciplinary consultation meeting, and the decision was made to proceed with concurrent chemoradiotherapy.

The initial clinical examination showed a patient who was hemodynamically and respiratory stable, apyretic, with a blood pressure of 124/83, heart rate of 87 beats per minute and respiratory rate of 28 breaths per minute. A neurological examination was then performed and revealed a patient with a Glasgow Coma Scale (GCS) score of 15, equal and reactive pupils, no neck stiffness, able to stand and walk without assistance and able to hold the Barre and Mingazzini tests. Muscle strength was rated at 5/5 in all four limbs, with preserved deep and superficial sensations. The deep tendon reflexes were present and symmetrical, and the examination of the cranial nerves revealed the presence of Parinaud’s syndrome.

An ophthalmologic examination revealed a visual acuity at 1/10, bilateral papilledema at stage 2 on fundus examination. The rest of the somatic examination was normal.

**Figure 1.**
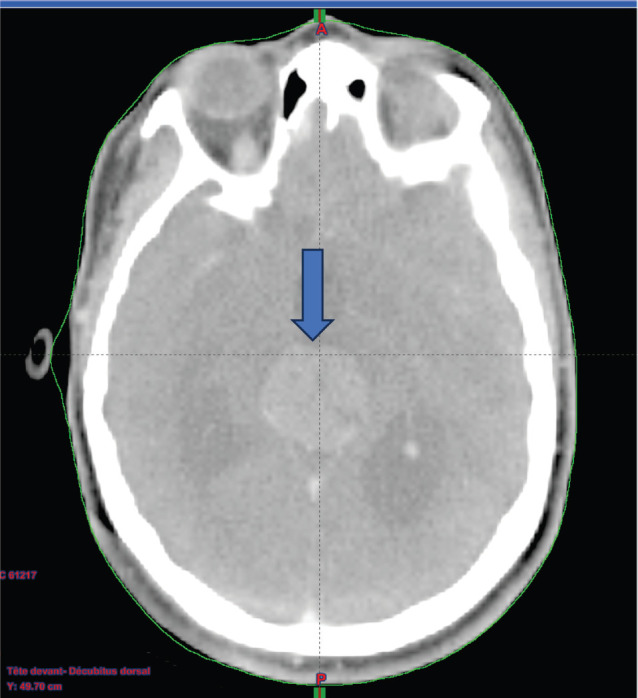
A contrast-enhanced brain CT scan reveals findings suggestive of a tumour in the pineal region.

A chemotherapy (CT) scan of the orbito-cerebral region (Figure 1) was performed and revealed the presence of a hypodense lesion in the pineal region, measuring approximately 3 cm in height, with an anteroposterior axis of 27 mm and a transverse axis of 25 mm. The lesion exhibits heterogeneous contrast enhancement, with an outer portion remaining hypodense. This lesion in the pineal region causes dilation of the third ventricle upstream, with mildly ecstatic lateral ventricles, but without visible transependymal resorption. Additionally, there were no visible parenchymal abnormalities, particularly no abnormalities in the posterior cranial fossa, and the fourth ventricle was not dilated.

**Figure 2.**
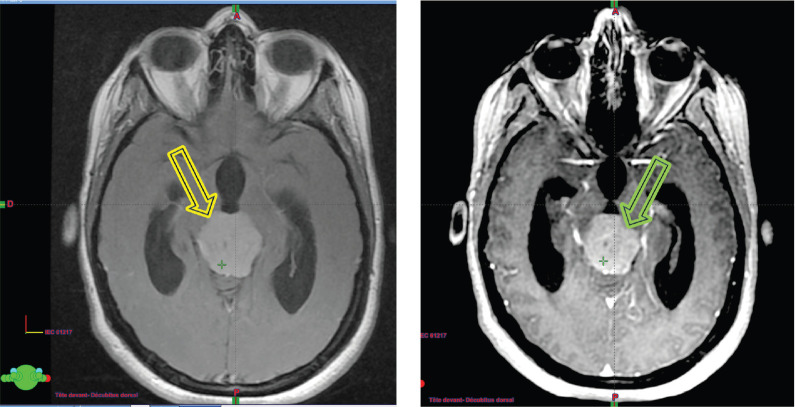
(a): The isointense tumour lesion in T2 flair. (b): Hyperintense in T1 Gado.

Upon her admission to the neurosurgery department at our institution, a brain MRI (Figure 2a and b) was performed, revealing an extra-axial supratentorial tissue mass centered on the pineal gland. The mass was roughly round in shape, well-defined, with lobulated contours, described as isointense on T1, T2 and FLAIR sequences compared to the gray matter. It contained a central area of hyperintense signal on T2, with restricted diffusion on diffusion-weighted imaging, and showed moderate and heterogeneous contrast enhancement after the injection of contrast agent, containing some vascular structures. No calcifications or hemosiderin deposits were observed on T2*. Spectroscopy revealed a peak of choline and a drop in N-acetyl-aspartate (NAA) (Choline/NAA ratio > 2). The mass measured 28 × 32 × 30 mm in diameter (APxTxH) and extended anteriorly, displacing the fourth ventricle, leading to bi-ventricular dilation (with measurements of 23 mm on the right and 26 mm on the left), showing signs of transependymal resorption. Inferiorly and posteriorly, it exerted pressure on the superior vermis, with a persistent layer of cerebrospinal fluid (CSF) separating it, and superiorly, it encompassed the two internal cerebral veins, which remained patent. The MRI also showed signs of increased intracranial pressure, characterised by flattening of the posterior poles of the eyeballs, thickening of the optic nerve sheaths and an arachnoidocele.

The cervico-dorso-lumbar MRI and CSF cytology were both negative, showing no pathological seeding.

Subsequently, the patient underwent a stereotactic biopsy, with the pathological and immunohistochemical study showing the following results: Synaptophysin: Positive; CD-117: Negative; GFAP: Negative; Ki67 labeling index: High (75%). These findings were consistent with a diagnosis of PB.

A brain CT scan performed 24 hours after the procedure revealed a lesion centered on the pineal gland, causing active bi-ventricular hydrocephalus, with an hematoma at the splenium of the corpus callosum, ruptured at the left ventricular level, leading to an intraventricular hemorrhage and flooding of the occipital horn of the left lateral ventricle.

A follow-up brain CT scan on day 7 showed a lesion that remained stable in size, still centered on the pineal gland, causing active tri-ventricular hydrocephalus and partial regression of the hematoma in the splenium of the corpus callosum, with ongoing left ventricular flooding.

Upon her admission on the day of the consultation in the RT department, the patient was conscious, with a GCS score of 15, and bilateral blindness, but without neck stiffness.

Subsequently, a dosimetric scan was performed using a 3-point mask and knee supports as positioning aids. Alignment using wall lasers, with intersection at the level of the tragus and transfer of images from the treatment-planning system to the contouring console.

RT was then planned, with a dose of 36 Gy to the craniospinal axis, and a boost of 18 Gy to the tumour.

The delineation of the craniospinal target volumes was performed with the adoption of variable margins [planning target volume (PTV) of 5 mm for the brain and cervicothoracic spine and PTV of 7 mm for the lumbosacral spine] to account for any positioning variability.

The first phase of the treatment was carried out using 6 MV photons, with three-dimensional conformational planning, utilising anteroposterior beams for the spine and two lateral beams for the brain. The dose distribution ranged between 95% and 107% according to ICRU 50 guidelines.

The second phase of the treatment was performed using 9 beams of 6 MV, with static intensity-modulated radiation therapy, prescribing 100% of the dose to the median of the volume. It was ensured that the 95% isodose covered at least 98% of the volume and that the 107% isodose did not exceed 2% of the volume, following the recommendations of ICRU 83.

During the treatment, the RT was well tolerated by the patient, with only mild nausea and tiredness (CTCAE Grade 2), both of which resolved with corticosteroid treatment.

A craniospinal MRI performed 3 months after the completion of RT showed significant regression of the pineal tumour, along with improvement in the hydrocephalus (estimated response of 38%).

Clinically, the patient no longer complains of headaches and shows no motor or sensory deficits, although she still has bilateral blindness.

The latest brain MRI performed 24 months after RT showed a stable appearance of the PBL compared to prior imaging.

To date, after 26 months of follow-up, the results of the exclusive RT treatment remain positive.

## Discussion

PBL, accounting for 24% to 50% of PPTs, is a rare malignant tumour classified among primitive neuroectodermal tumours. It primarily affects children, representing less than 1% of pediatric intracranial tumours and less than 0.1% of all intracranial tumours [4, 5]. Although rare, cases in adults, such as the one we report, have been observed. This aggressive tumour exhibits rapid progression with a high potential for leptomeningeal dissemination, making it a therapeutic challenge.

In our case, the diagnosis was made through imaging and confirmed histologically. Initial management of PBL typically involves a multimodal approach, combining surgery, RT and CT, especially when complete tumour resection is not feasible. However, the exact role of each modality, particularly surgery, remains a topic of ongoing debate in the literature, particularly in adults, where data are limited.

### Role of surgical resection

While the current consensus for pediatric PBL generally favours aggressive surgical resection as a first-line treatment to minimise the risk of tumour recurrence, the data regarding the impact of gross total resection (GTR) or subtotal resection (STR) tumour resection on overall survival (OS) in adults remains limited [6]. In the literature, treatment strategies vary, with approaches ranging from surgery alone to RT alone or in combination with CT [6]. Due to the low number of reported adult cases of PBL, optimal management remains a subject of controversy. However, in the United States, the current standard treatment for adult patients with PBL involves a multimodal approach combining surgery, RT and CT [6].

In our case, although surgical resection could not be performed, the combination of RT and CT was not an option due to the patient’s complete refusal of any cytotoxic treatment. Exclusive RT became our only and ultimate hope to reduce the tumour size and stabilise the disease.

Some studies suggest that there is no clear relationship between the extent of resection and OS in PBL. Pusztaszeri *et al* [6] studied 42 patients who underwent both surgery and RT, and found no statistically significant benefit in terms of OS with larger resection margins. Similarly, Mallick *et al* [15] demonstrated no association between OS and the extent of resection. However, other studies have reported contradictory results. Lee *et al* [8] showed an improvement in OS for patients who underwent total tumour resection in a cohort of 34 adult PBL patients. Another study by Greppin *et al* [9] found that patients who did not undergo surgical treatment had a lower OS compared to those who received surgery, regardless of its type.

A recent article published in December 2024 by Chu *et al* [14] is particularly noteworthy. It is a systematic review of adult PB, including 108 patients from all published case reports of histologically confirmed adult PBL, written in English and published before June 2021. The aim of this review was to evaluate the relationship between 5- and 10-year OS and various prognostic factors (age, gender, surgery, RT and CT). The study found a significant relationship between the quality of surgery and OS for this histological type [14].

### Role of adjuvant treatment

#### Radiation therapy

The role of postoperative treatment in PBL remains undetermined; however, some reported cases have shown that RT helps control the tumour and improve survival in patients with PBL. A study of Mehkri *et al* [10] on the SEER database, published in 2023, including 201 adult patients with PBL, found that RT with or without surgery was associated with an improvement in 5-year OS compared to the absence of RT (77.3% versus 63.2%, *p* = 0.020).

We can also refer to the study by Lee *et al* [11], conducted on 34 adults aged 16 years or older, who were diagnosed with PBL between 1969 and 1998. They found that the median survival of patients who received a dose of ≥ 40 Gy of cranial irradiation was three times higher than that of patients receiving lower doses (29.8 versus 8.1 months). However, no prospective study has yet validated the efficacy of RT or determined the optimal doses for the treatment of PBL.

A similar observation was made in the study by Chu *et al* [14] where RT not only showed statistical significance in the Kaplan-Meier curve and univariate Cox analysis (*p* < 0.001), but also appeared as the only independent prognostic factor in the multivariate Cox analysis.

Regarding the impact of the type of RT, this study attempted to explore the effect of boost + craniospinal irradiation (CSI), focal irradiation and CSI alone on survival. Although no statistical difference in the type of RT was found, CSI + boost showed a trend toward better survival. However, to date, there is no retrospective analysis of adult PBL that explores this factor [14].

#### Chemotherapy

The role of CT remains unclear for adult patients and is primarily derived from studies on medulloblastomas. In our patient, CT was not administered, which is why we opted for exclusive RT. In the study by Chu *et al* [14] the Kaplan-Meier curve and univariate Cox analysis showed that CT was beneficial and significantly improved survival. However, the multivariate Cox analysis revealed that CT could not be used as an independent prognostic variable. This suggests that CT, when combined with surgery and RT, may improve survival rates [14]. Brandes *et al* [16] propose that patients should be stratified based on the risk of recurrence when planning adjuvant treatment. In any case, further studies are needed to address the question of the best CT regimen.

## Conclusion

The case we reported is a rare and aggressive disease that affected a patient in her third decade of life. Despite the rarity of evidence, data and the lack of a standardised protocol for exclusive RT treatment, this case demonstrates the remarkable success of RT in treating and stabilising the tumour in this young patient. Unfortunately, she couldn’t regain her normal life due to her blindness, but at least she was able to rid herself of the headaches.

We can conclude that RT, on its own, remains a miraculous tool that can save the lives of patients in whom surgical resection of the tumour was not performed.

Further studies, both diagnostic and therapeutic, are needed in order to develop a standardised protocol for this highly malignant and aggressive type of CNS tumour.

## Conflicts of interest

The authors declare that they have no known competing financial interests or personal relationships that could have appeared to influence the work reported in this paper.

## Funding

This case report has received no financial support. Regarding publication, we hope that ecancer will assist in its publication

## Author contributions

OA.S: Writing – original draft, Writing – review and editing.

A.FZ: Writing – review and editing, Data curation.

KS: Writing – review and editing, Conceptualisation

F.FZ; A.Z and BT : supervision and guidance»

## Figures and Tables

**Figure 3.**
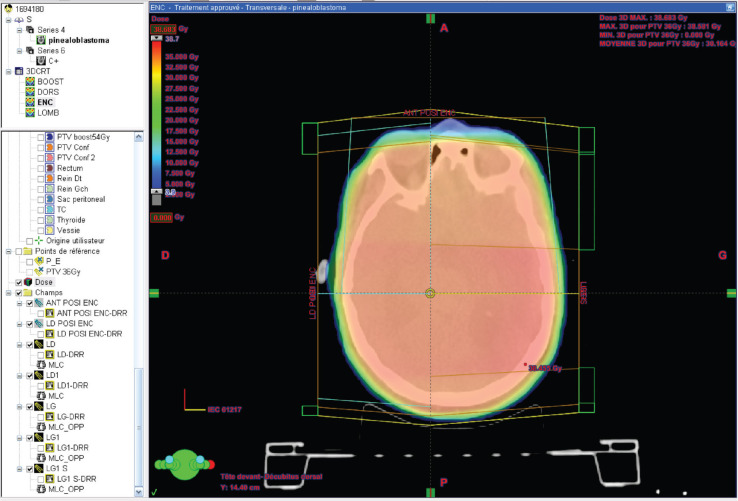
Dose distribution to the brain 36 GY in 3DCRT*. *3DCR : tree dimensional conformal radiation therapy.

**Figure 4.**
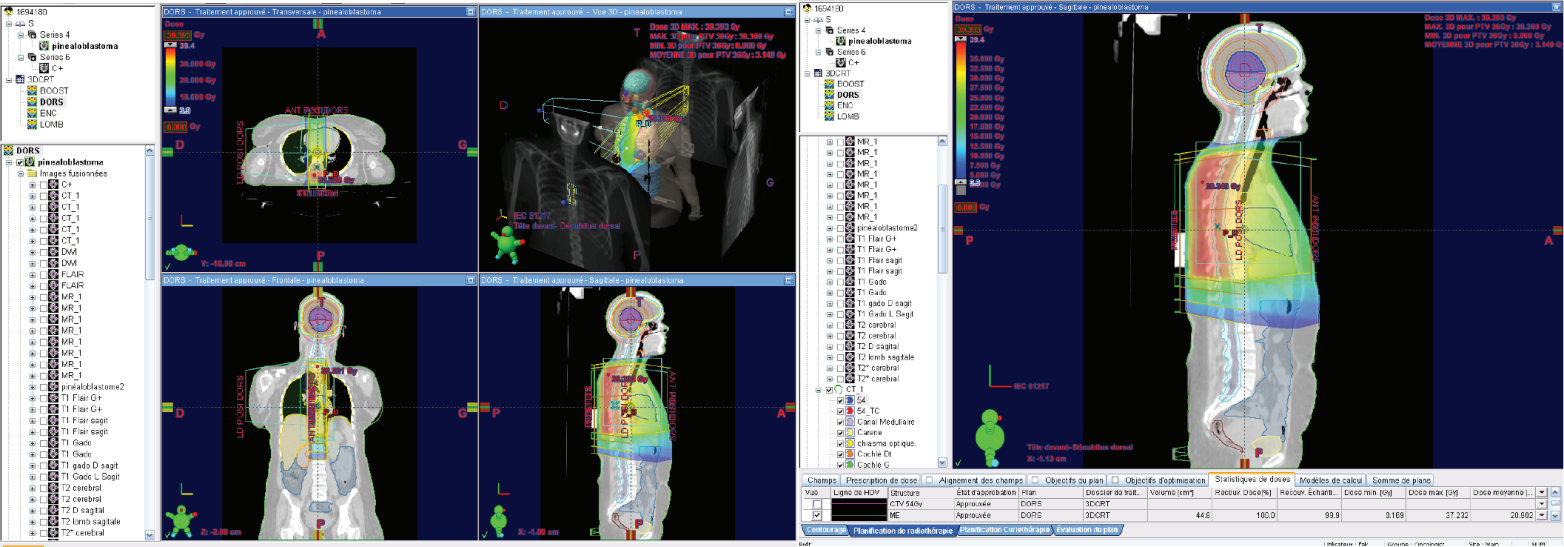
Dose distribution to the 36 GY thoracic spine in 3DCRT.

**Figure 5.**
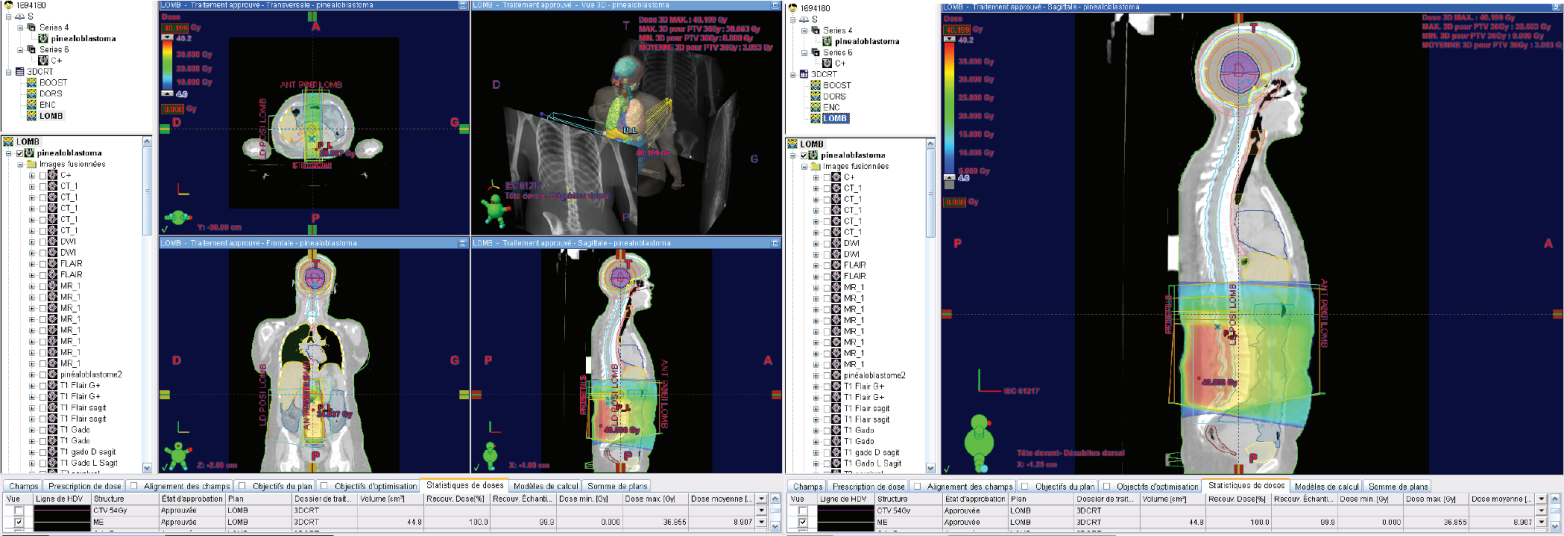
Dose distribution to the lumbar spine 36 GY in 3DCRT.

**Figure 6.**
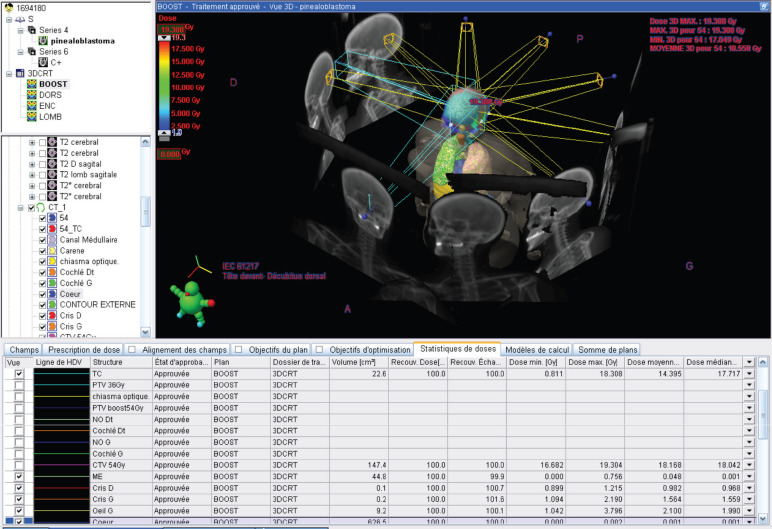
18 GY BOOST in static IMRT* with constraints on organs at risk. IMRT*: Intensity Modulated Radiation Therapy.

**Figure 7.**
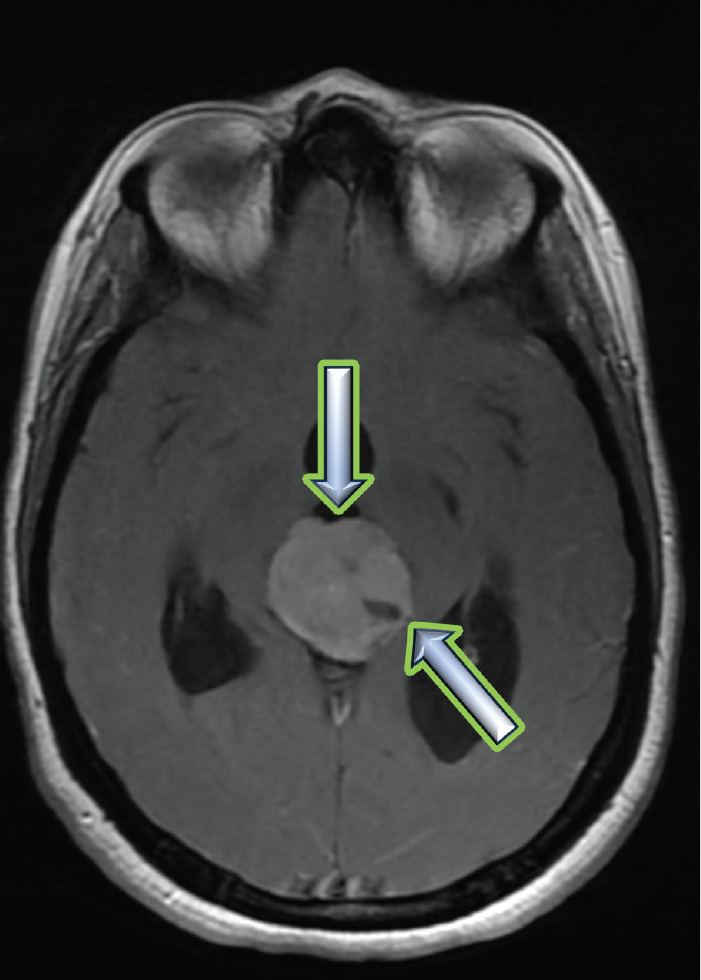
Brain MRI 3 months after RT treatment, showing an estimated response of 38% on a T2 flair sequence.

**Figure 8.**
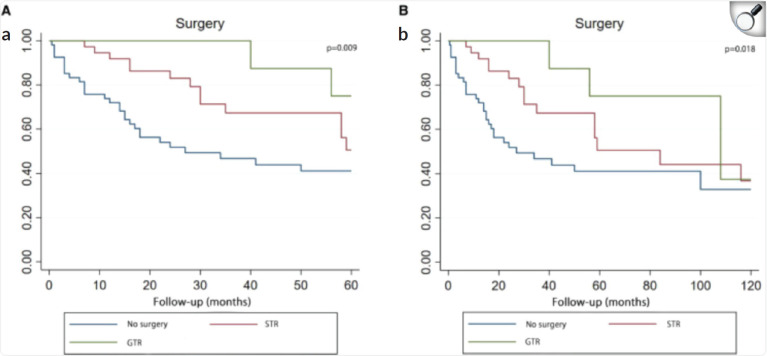
Kaplan–Meier curve analysis (Log-rank test) illustrating patient survival rates (n = 107) between GTR, STR and no surgery for 5 years (a) or 10 years (b). STR, subtotal resection; GTR, gross total resection.

**Figure 9.**
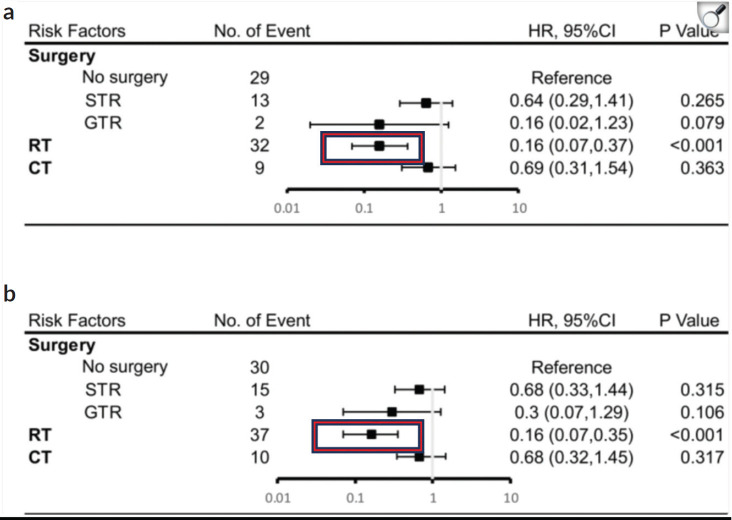
Results of multivariate analysis on OS at 5 years (a) or 10 years (b). HR, hazard ratio; CI, Confidence Interval. *Statistically significant. STR, subtotal resection; GTR, gross-total resection; RT, radiotherapy; CT, chemotherapy [14].

**Figure 10.**
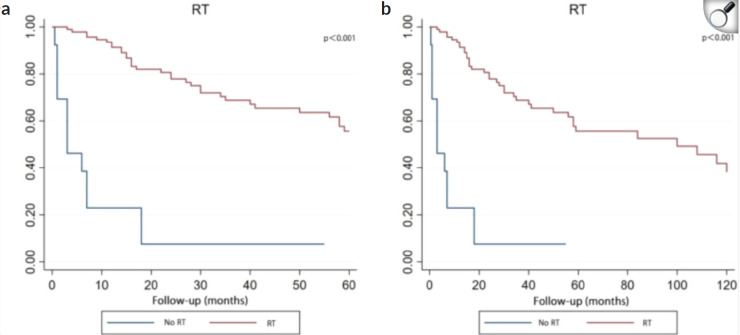
Kaplan–Meier curve analysis (Log-rank test) illustrating the survival rates of patients (n = 108) between RT and no RT for 5 years (a) or 10 years (b). RT, radiotherapy [14].
